# Prioritizing embryologist health: occupational health hazards and preventive strategies

**DOI:** 10.3389/fpubh.2026.1867249

**Published:** 2026-06-17

**Authors:** Man-Xi Jiang, Shao-Qing Chen, Huai L. Feng, Chang-Long Xu, Yan Zhu

**Affiliations:** 1 Center for Reproductive Medicine, The Affiliated Guangdong Second Provincial General Hospital of Jinan University, Guangzhou, China; 2 New York Fertility Center, New York-Prebyterian Healthcare System Affiliate Weill Cornell Medical College, New York, NY, United States; 3 Guangxi Clinical Research Center for Reproductive Medicine, Nanning Second People's Hospital/The Third Affiliated Hospital of Guangxi Medical University, Nanning, China; 4 Department of Immunology, University of Pittsburgh, Pittsburgh, PA, United States

**Keywords:** burnout, clinical embryologist, ergonomics, laboratory safety, musculoskeletal disorders, occupational health, preventive strategy

## Abstract

Clinical embryologists are pivotal to assisted reproductive technology (ART) success, performing critical procedures from gamete manipulation to embryo transfer, yet they face multifaceted and under recognized occupational health challenges in a high-stakes environment. This review examines a variety of occupational risks and diseases affecting embryologists, synthesizing literature on physical, psychological, and ergonomic hazards. Musculoskeletal disorders are prevalent, with shoulder pain increasing in a dose-dependent manner as career length progresses. Psychological morbidity is similarly significant, as a subset of embryologists have stress-related mental health issues and elevated rates of emotional exhaustion. Furthermore, gaps in safety compliance persist, including insufficient protection during the handling of semen or liquid nitrogen. This review ultimately offers two concrete contributions: (1) reframing embryologist health as a quality assurance imperative rather than solely a personnel issue, and (2) leveraging automation and digital technology as a targeted strategy to reduce ergonomic and psychological hazards. Building on this framework, this review also proposes a multi-level intervention strategy encompassing individual, ergonomic, organizational, and technological dimensions to alleviate the aforementioned risks, directly linking embryologist well-being to treatment safety and clinical outcomes.

## Graphical abstract

**Figure:**
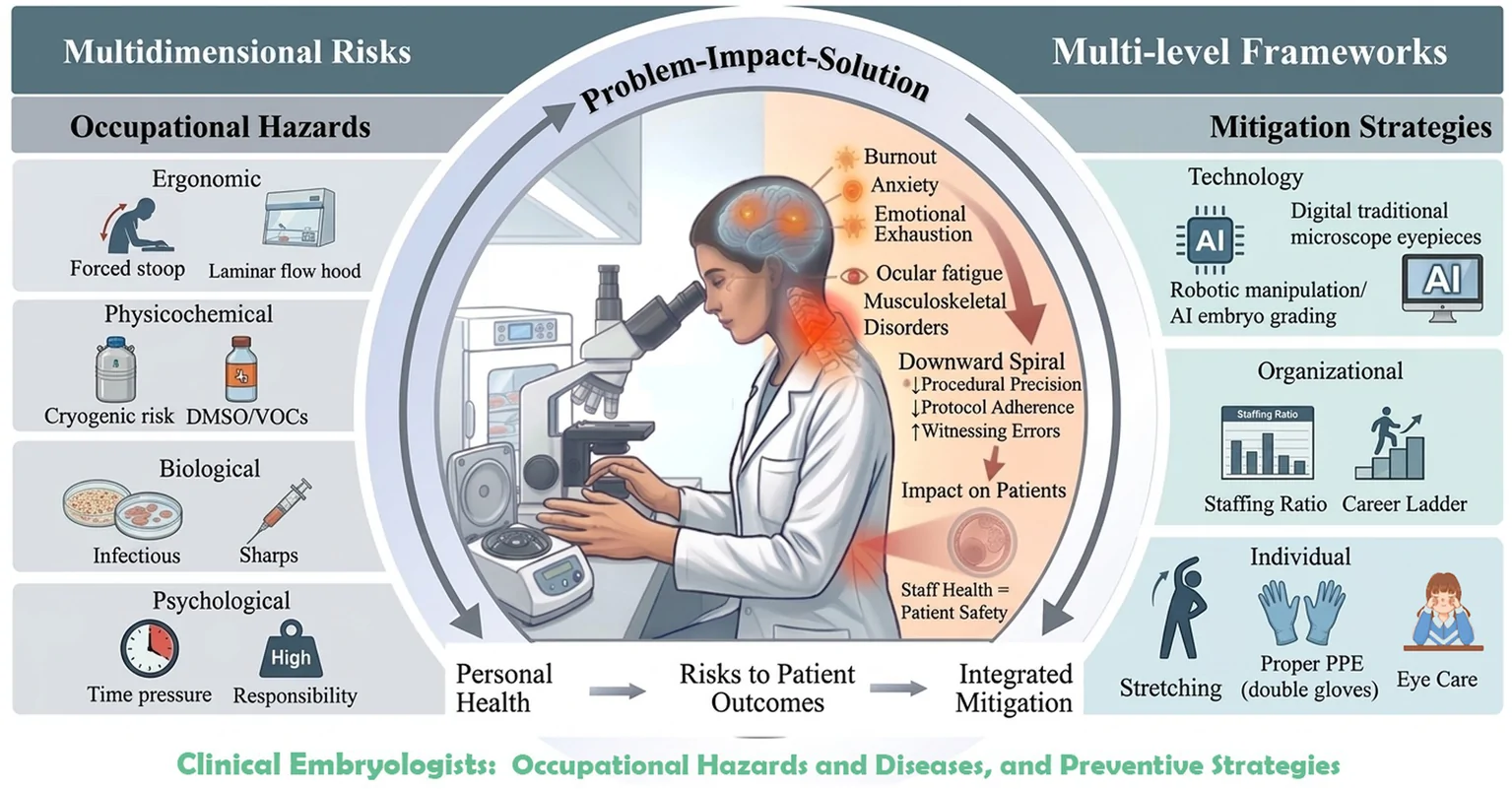
Occupational health hazards and intervention strategies for clinical embryologists. This graphical abstract illustrates the multi-dimensional relationship between laboratory-specific hazards, their physiological and psychological impacts, and the proposed paradigm shifts for improving clinical outcomes. The left panel categorizes occupational hazards into four domains, namely ergonomic, physicochemical, biological, and psychological. The middle panel highlights resulting health issues (e.g., musculoskeletal disorders and emotional exhaustion) and the burnout–error cycle, where reduced concentration leads to clinical errors, which in turn increase stress and exacerbate burnout. The paradigm shift panel proposes three pillars: recognizing embryologist health as key to quality assurance, modeling the burnout–error relationship, plus leveraging automation and digital technologies to mitigate physical risks. The right panel outlines a four-pronged intervention strategy spanning individual, ergonomic, organizational, along with technological levels.

## Introduction

Clinical embryologists hold a position of profound responsibility, serving as the specialized scientist tasked with the delicate gamete manipulation, fertilization, culture, and the subsequent transfer or cryopreservation (1). Their work requires sustained static working postures, microscopic precision, unwavering attention to detail, and the emotional resilience to manage clinical outcomes that profoundly impact patients’ lives.

Despite the critical nature of their role, the occupational health of embryologists has historically received limited attention from researchers, laboratory directors, and regulatory bodies (2). The well-being of clinical embryologists remains a largely neglected aspect in the field of reproductive medicine (3). This oversight is particularly concerning, given accumulating evidence that embryologists experience a disproportionately high burden of work-related illness compared to other healthcare professionals (4). Such an occupational health deficit not only affects individual well-being but may also pose risks to ART laboratory quality and patient safety by influencing procedural precision, decision-making, and safety compliance (5).

Since 1978, the field of embryology has evolved from a niche specialization into a robust global profession that executes millions of ART cycles each year (6, 7). As ART volumes have increased and laboratory techniques have grown more complex, the physical and psychological stress imposed on embryologists have intensified correspondingly (8).

This issue extends beyond individual well-being, because emerging evidence suggests that embryologist burnout and occupational stress directly correlate with laboratory performance metrics, including witnessing discrepancies, protocol adherence, and potentially clinical outcomes such as fertilization and pregnancy rates (5). Thus, protecting embryologist health is inseparable from ensuring patient safety and maintaining quality standards in ART laboratories.

This review provides a comprehensive overview of occupational health issues affecting embryologists and challenges the prevailing assumption that embryologist well-being is solely an individual or human resources concern by systematically linking occupational health metrics to laboratory quality indicators, such as witnessing discrepancies and protocol adherence. It also synthesizes emerging evidence on the dose-dependent relationship between cryostorage-related anxiety and burnout risk, thereby identifying a previously underappreciated psychological hazard unique to contemporary ART practice. Furthermore, it proposes a conceptual shift: viewing the embryologist as a critical operator and safety-critical professional whose occupational health directly conditions patient safety and treatment outcomes. Under this reconceptualization, laboratory equipment design and facility layout must be optimized for the operator—accounting for ergonomic demands, cognitive load, and fatigue risk—rather than expecting the embryologist to adapt indefinitely to existing infrastructure.

## Qualification requirement, practice scope, and work environment for embryologists

### Professional qualification

Entry into the clinical embryology profession typically necessitates a bachelor’s degree in biological sciences, although the increasing complexity of the field has led many practitioners to pursue master’s or doctoral degrees (9). Formal training pathways vary substantially by jurisdiction, ranging from structured academic certificate programs to informal apprenticeship-based models. Yet conspicuously absent from these competency frameworks is any standardized requirement pertaining to occupational health literacy, ergonomic training, or fatigue risk management—despite emerging evidence that these factors directly correlate with laboratory safety and clinical outcomes (5). This absence reflects a broader pattern: professional entry standards prioritize technical proficiency in gamete and embryo handling while treating embryologist well-being as a peripheral, rather than integral, component of laboratory quality assurance.

In the United States, the American Society for Reproductive Medicine (ASRM) has established minimum standards requiring embryologists to possess a bachelor’s or master’s degree in a chemical, physical, or biological science from an accredited institution (9). Candidates must document the completion of a minimum of 30 ART procedures under supervision, accompanied by a formal attestation of competency from the training laboratory and a commitment to 24 h of continuing education every 2 years. Senior-level roles require a minimum of 3 years of experience and the performance of at least 20 annual procedures to maintain technical proficiency, while laboratory supervisors face more stringent mandates, including graduate-level education and extensive documented experience (9). Notably, nowhere in these ASRM standards do requirements appear for training in workplace ergonomics, recognition of burnout symptoms, or institutional policies for workload limits—despite embryologists reporting musculoskeletal pain rates exceeding 45% (2) and burnout rates approaching 60% (4).

Similarly, China operates under the Technical Specifications for Human Assisted Reproductive Technology (2003) issued by the National Health Commission. Laboratory technicians must hold a degree in medicine or life sciences and complete 3 months of systematic training at a government-approved ART base. Upon passing a formal assessment, they receive a Certificate of Training and must be registered within a licensed healthcare institution. At the leadership level, a Laboratory Director must hold a Doctorate (PhD) or a Senior Professional Title, demonstrating expertise in clinical embryology and genetics alongside specialized training at a designated national center to ensure rigorous oversight and ethical compliance. Critically, the Technical Specifications (2003) make no mention of occupational health protections, mental health support, or ergonomic standards for laboratory personnel. This regulatory silence persists despite China operating over 500 ART centers and performing an estimated one million IVF cycles annually—environments where embryologists face identical physical and psychological stressors as their Western counterparts, yet with arguably less explicit regulatory attention to their occupational well-being (10).

The variation in training pathways across jurisdictions has direct—and underappreciated—implications for occupational health standardization. Structured academic programs (e.g., UK’s HCPC-registered clinical scientist training) typically include formal instruction in laboratory safety, reflective practice, and professional boundaries—components that may indirectly promote self-awareness of occupational health risks. In contrast, apprenticeship-based models often embed trainees in existing laboratory cultures that may normalize long hours, understaffing, and ergonomic neglect. This disparity creates a two-tiered occupational health landscape without explicit acknowledgment: embryologists trained in structured pathways may enter the workforce with greater preparedness to identify and advocate for safe working conditions, while those trained via apprenticeship risk internalizing hazardous practices as routine. The absence of harmonized occupational health training in ART field not only perpetuates geographic inequities in worker protection but also undermines efforts to establish universal laboratory safety standards. A minimal harmonization standard would require three elements to be integrated into all embryology training pathways, regardless of jurisdiction: (1) ergonomic principles applied to micromanipulation workstations; (2) recognition and reporting of burnout symptoms; and (3) workload limit policies derived from human factors research rather than arbitrary clinic scheduling.

A striking disconnect emerges when comparing the specificity of patient safety regulations versus occupational health provisions for embryologists. ART laboratory regulations—whether CAP/TJC accreditation in the US (9), EUTCDs in Europe (11, 12), or HFEA licensing in the UK (13–15)—mandate rigorous standards for equipment calibration, temperature monitoring, traceability, and witnessing protocols. Yet they remain largely silent on the corollary: human operators can also fail, particularly when fatigued, stressed, or in pain. The UK HFEA’s Code of Practice, for example, runs to hundreds of pages detailing everything from air quality standards to cryostorage contingency planning (16). Nowhere does it mandate maximum shift durations, minimum rest breaks between procedures, or ergonomic assessments of ICSI workstation. Similarly, ESHRE’s voluntary certification focuses on staff competency and KPIs through triennial inspections (6, 11, 12), yet its criteria do not include staff burnout monitoring or workload metrics. This regulatory asymmetry—meticulous attention to equipment reliability paired with minimal attention to operator reliability—represents a critical blind spot in contemporary ART governance.

### Core technical responsibilities

Clinical embryologists specialize in the handling and culture of human gametes and embryos for ART procedures. Unlike many medical laboratory professionals who analyze patient samples and generate diagnostic reports, embryologists perform both diagnostic and therapeutic functions, directly manipulating the biological materials that will be transferred to patients (17).

The scope of embryology practice encompasses a comprehensive array of technical procedures. It begins with semen processing, involving the assessment of sperm concentration, motility, and morphology, alongside preparation for conventional *in vitro* fertilization (IVF) or intracytoplasmic sperm injection (ICSI). This is followed by oocyte handling, including retrieval from follicular fluid, nuclear maturity assessment, and preparation for insemination or cryopreservation. Fertilization is then executed via IVF or ICSI, with assessment typically occurring 16–18 h post-insemination. Subsequent stages include embryo culture, which demands rigorous incubation management and media preparation to maintain optimal preimplantation conditions, and embryo selection for transfer or cryopreservation, where morphology is evaluated at critical stages to identify high-implantation potential. Finally, the field mandates expertise in cryopreservation (vitrification and warming) and cryo-storage management, requiring meticulous inventory control, liquid nitrogen maintenance, and specimen documentation.

### Work environments

The embryology laboratory is a unique environment characterized by strictly controlled parameters including temperature, humidity, and air quality, that optimize embryo development but may compromise staff comfort (18). Work in the IVF laboratory is heavily reliant on microscopes, demanding sustained awkward postures and continuous intense visual concentration (19, 20); the administrative burden of documentation, proficiency testing, and audit preparation consumes substantial time and is a documented contributor to workload stress and professional burnout (3, 21).

These stressors are compounded by the high-stakes nature of the work, where errors such as gamete loss, specimen mismatching, or pregnancy failure, impose a heavy psychological burden (22). Finally, the field mandates irregular hours and on-call responsibilities, as ART cycles are dictated by biological timing rather than standard business hours, often disrupting work-life balance (21).

## Occupational diseases from physical hazards to psychological risks

**Figure 1:**
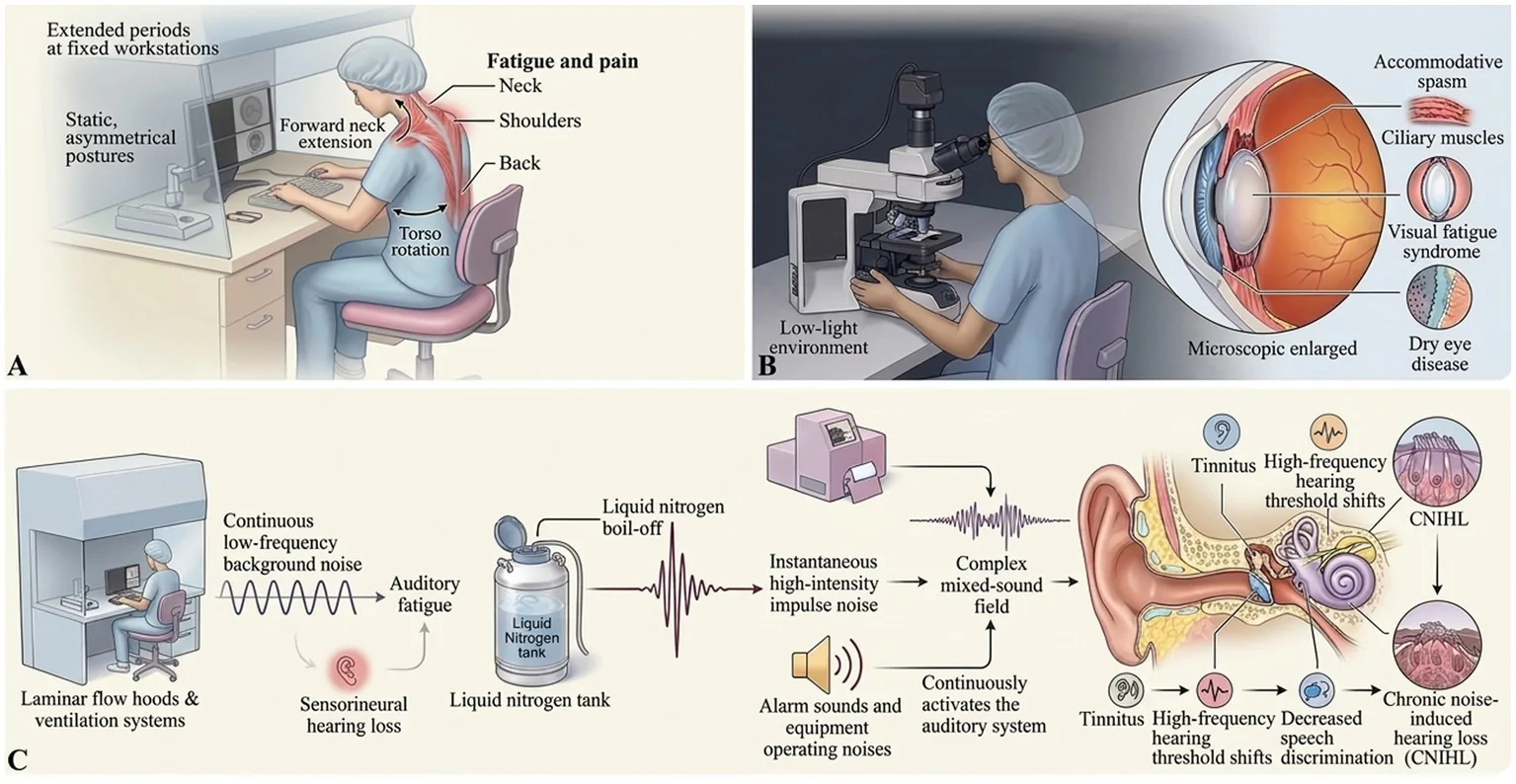
Common physical diseases of clinical embryologists. This diagram illustrates occupational health risks for embryologists. **(A)** Musculoskeletal Strain: Prolonged static postures at fixed workstations characterized by cervical flexion and axial torso rotation that precipitate chronic fatigue and localized pain in the neck, shoulders, and lumbar region. **(B)** Ocular Pathologies: Extended microscopic work in low-light environments exacerbates the risk of accommodative spasms, visual fatigue syndrome, and dry eye disease. **(C)** Auditory Hazards: The laboratory’s complex acoustic environment presents significant risks. This includes continuous low-frequency noise from laminar flow hoods and ventilation, alongside transient high-intensity impulse noise from liquid nitrogen boil-off. Long-term exposure to this mixed-sound field may lead to tinnitus, high-frequency hearing threshold shifts, and reduced speech discrimination, potentially progressing to permanent noise-induced hearing loss (NIHL).

Embryologists are exposed to a unique and array of physical occupational hazards that arise from the distinctive demands of their work environment. Unlike many laboratory settings where chemical or biological exposures dominate safety considerations, the embryology laboratory presents a complex interplay of ergonomic, sensory, and physical stressors that collectively impact practitioner health and well-being. These occupational challenges arise from multiple interconnected sources: sustained awkward postures during prolonged micro-manipulation under sterile conditions; intense visual demands from extended microscope use; exposure to potentially infectious biological materials; contact with cryoprotectants and other systemically active chemicals; and physical hazards such as cryogenic burns and sharps injuries. The cumulative burden of these occupational exposures has been increasingly recognized as a significant concern for workforce sustainability, with surveys documenting substantial prevalence of work-related health complaints among embryologists. Table 1 summarizes key physical occupational diseases causing musculoskeletal, ocular, and auditory disorders (Table 1 and Figure 1). Table 2 extends to encompass biological hazards, chemical exposures, and physical hazards encountered in the ART laboratory setting.

**Table 1:** Characteristics of physical occupational diseases.

Occupational diseases	Prevalence	Biomechanical mechanisms and risk factors
MSDs	MSDs affect 45.3% of embryologists, with dose-dependent correlation to career length (2).	Cumulative trauma from microscopy, awkward postures, and prolonged standing causes soft tissue ischemia via sustained cervical flexion (20–40°), shoulder abduction, wrist extension/ulnar deviation, and static paraspinal loading
Ocular problems	Over 3% of embryologists have ocular problems (2).	Microscopy demands sustained accommodation/convergence for 100–150 μm structures; constant low-light refocusing requires near-maximal resolution, causing continuous strain.
Auditory problems	3% of embryologists have noise-induced auditory issues, including progressive threshold shifts (2).	Noise (liquid nitrogen, alarms, ventilation) causes sustained physiological stress and risk of progressive hearing threshold shifts.

MSD, Musculoskeletal disorder.

**Table 2:** Characteristics of occupational hazards.

Occupational hazards	Specific hazard/source	Key risks	References
Biological hazards	Infectious materials (semen, follicular fluid, blood, tissues)	Exposure to blood-borne pathogens (HIV, HBV, HCV) and sexually transmitted infections.	(23, 36, 37)
	Semen processing	Highest risk procedure; raw ejaculate may contain high viral loads. Needlestick injury incidence >3% (UK survey).	(2)
	Follicular fluid handling	Risk from aspirates containing blood. 19.6% of embryologists never wear gloves (Spanish survey).	(33, 34)
	Cryopreservation	Liquid nitrogen can be a vector for cross-contamination if infected specimens leak (e.g., HBV transmission documented).	(35)
	Waste handling	Risk of sharps injuries from contaminated consumables.	
	Patient population and PPE adherence	Many infected individuals are unaware of their status. Despite universal precautions, PPE adherence is inconsistent (e.g., 30% rarely/never use liquid nitrogen protection).	(35, 36, 39, 40)
Chemical exposures	Culture media	Potential skin or respiratory irritation with repeated exposure.	
	Cryoprotectants (DMSO, ethylene glycol, propylene glycol)	Can penetrate intact skin; potential systemic effects with chronic exposure.	(34, 42, 43)
	Cleaning agents	May release volatile organic compounds or cause respiratory sensitization.	
	Carbon dioxide (CO₂)	Asphyxiation risk in confined spaces (mitigated by ventilation).	
	Knowledge gap	Poorly understood effects of chronic low-level chemical exposure on health.	
Physical hazards	Liquid nitrogen	Cryogenic burns from extreme cold (−196 °C); incidence >3% (UK survey).	(2)
	Sharps (ICSI needles, biopsy pipettes)	Risk of infectious disease transmission and local trauma (inoculum volume small but risk not eliminated).	(41)
	Radiation/lasers	Lasers used for assisted hatching or biopsy present potential eye hazards if improperly shielded.	

HIV, Human immunodeficiency virus; HBV, Hepatitis B virus; HCV, Hepatitis C virus; PPE, Personal protective equipment; DMSO, Dimethyl sulfoxide; ICSI: Intracytoplasmic sperm injection.

### Physical occupational diseases

#### Musculoskeletal disorders

Musculoskeletal disorders (MSDs) represent the most prevalent physical occupational health issue affecting embryologists (Figure 1A), with a comprehensive survey of UK embryologists by Priddle et al. finding that 45.3% of respondents reported work-related musculoskeletal problems, making MSDs the single most common occupational health complaint (2). Symptom distribution reflects the specific physical demands of embryology work: shoulder pain and injury show particularly strong associations with career length, demonstrating a dose-dependent increase in incidence with years of service, suggesting cumulative trauma from sustained awkward postures (2); neck and upper back pain are also commonly reported, attributable to prolonged forward flexion while working at microscopes and laminar flow hoods, where standard equipment ergonomics often force embryologists to adopt positions that strain cervical and thoracic musculature (23); and lower back pain affects those who spend extended periods standing at laminar flow hoods for procedures such as oocyte retrievals or embryo culture, which must often be performed standing to maintain sterility and access equipment (24, 25) (Figure 1A). The biomechanical basis for these disorders can be understood through analysis of typical work postures: during microscopic examination, embryologists must maintain cervical flexion of 20–40 degrees to view through eyepieces, shoulder abduction and elevation to position arms at the microscope stage, wrist extension and ulnar deviation during micromanipulation, and static loading of paraspinal muscles to maintain seated position-postures maintained for hours daily that create sustained muscle tension, reduce blood flow to soft tissues, and accelerate degenerative changes in spinal structures (26). Notably, the introduction of ICSI in the 1990s, while revolutionizing treatment for male factor infertility, may have exacerbated these ergonomic risks, as it requires even greater precision and prolonged static positioning than conventional IVF, with individual injection procedures being more labour-intensive and time-consuming (8, 22, 27, 28).

#### Ocular problems

Visual strain and ocular symptoms represent another significant physical complaint among embryologists (Figure 1B); the UK survey identified ocular problems as occurring in more than 3% of respondents, though this likely underestimates the true prevalence given the visual demands of the work (2). Embryologists face multiple sources of visual stress in their daily practice, including prolonged microscope use requiring sustained accommodation and convergence that leads to eye fatigue and asthenopia (2, 29), frequent shifting of gaze between microscope eyepieces, laboratory monitors, and macroscopic work surfaces that constantly taxes ocular accommodation mechanisms, reduced ambient lighting and green-filtered microscope illumination implemented to protect embryos from light exposure which paradoxically increases visual effort (2, 29), and the inherently small target sizes of gametes and embryos measuring 100–150 micrometers in diameter, which demand near-maximal visual resolution and contribute to eye strain.

#### Auditory problems

While less documented, auditory issues affect a subset of embryologists, with the UK survey finding auditory problems exceeding 3% incidence among respondents (2). Several sources of potentially hazardous noise exist in ART laboratories, including liquid nitrogen handling where the filling of dewars and storage tanks produces loud boiling and hissing sounds that can exceed safe exposure levels with prolonged exposure as well as equipment alarms from incubators, cryo-storage monitors, and laboratory information systems, which may be both loud and psychologically stressful. Additional contributors include ventilation systems, where the high-efficiency particulate air (HEPA) filtration and positive pressure requirements for cleanroom operation generate continuous background noise, and the cumulative effect of traffic and conversation from multiple personnel working in confined laboratory spaces (Figure 1C). While noise levels in most embryology laboratories likely remain below occupational exposure limits that would cause hearing loss, the combination of noise with high cognitive demands may increase stress and reduce performance (2, 30).

### Infectious and physicochemical risks

**Figure 2:**
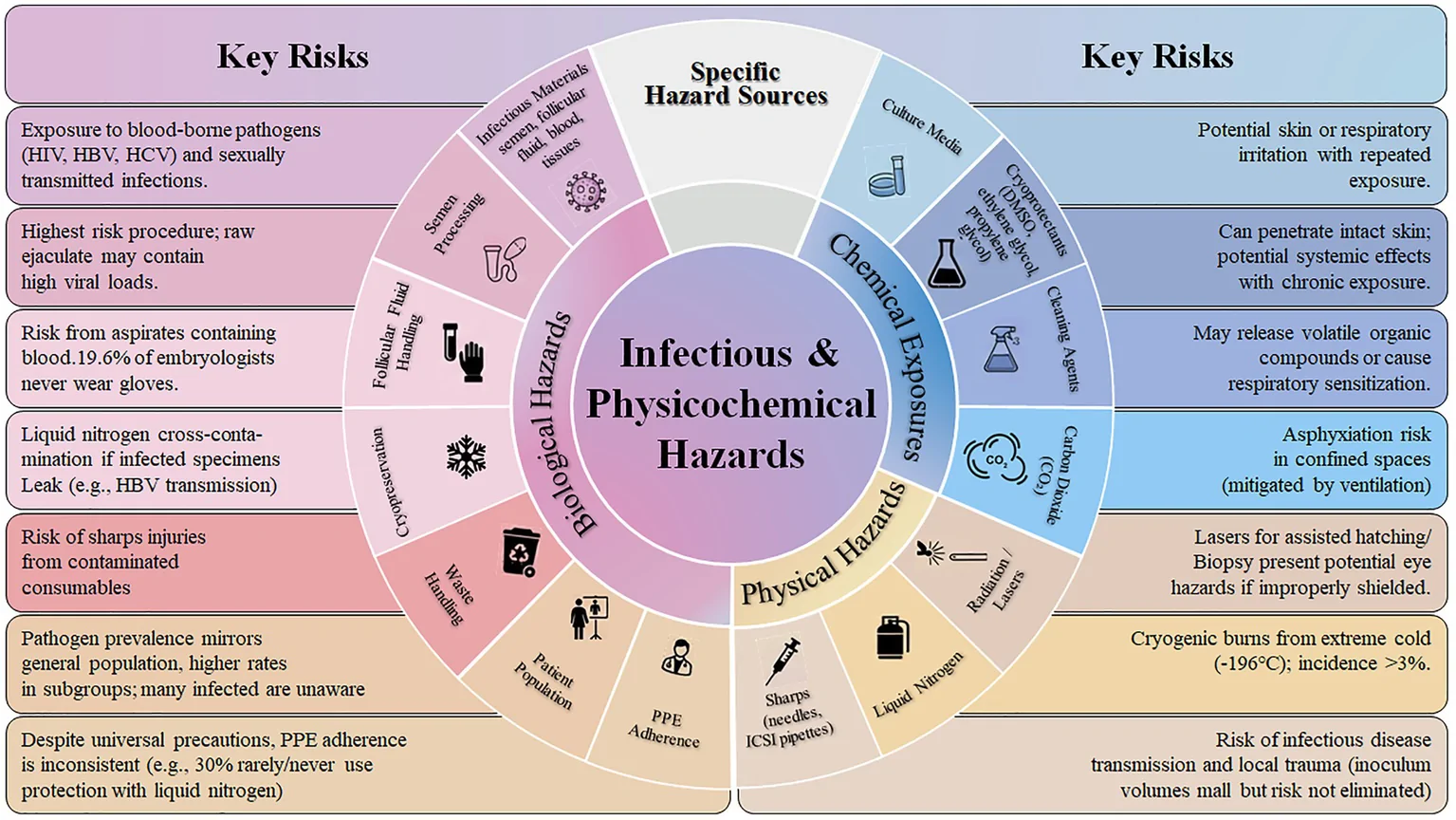
Infectious and physicochemical hazards for clinical embryologists. The schematic classifies laboratory risks into three primary domains including biological hazards, chemical exposures, and physical hazards. Biological hazards highlight the risks associated with handling infectious materials and the potential for blood-borne pathogen transmission, exacerbated by inconsistent PPE adherence. Chemical exposures delineate risks from culture media, cry protectants, cleaning agents, and CO2 levels, which can lead to skin irritation, respiratory sensitization, or systemic effects. Physical hazards encompass risks from radiation/lasers, liquid nitrogen, and sharps injuries. The outer panels detail specific key risks and prevalence data, emphasizing the critical need for standardized safety protocols and consistent use of personal protective equipment.

Handling semen and oocytes remains a critical biological safety vulnerability in laboratory safety. These are compounded by physical hazards, such as cryogenic burns and sharps injuries, which present acute trauma risks. Furthermore, chronic exposure to volatile disinfectants and cryoprotectants poses insidious long-term health consequences (Figure 2).

#### Biological infectious risks

Embryologists face daily exposure to potentially infectious biological materials, including semen, follicular fluid, blood, and other tissues, which may harbor blood-borne pathogens such as human immunodeficiency virus (HIV), hepatitis B virus (HBV), hepatitis C virus (HCV), and other sexually transmitted infections (31, 32). Different procedures carry varying levels of exposure risk: semen processing presents the highest risk, as raw ejaculate may contain high viral loads, and the UK survey identified needlestick injuries as a specific occupational hazard with incidence exceeding 3% (2); follicular fluid handling involves aspirates containing both blood and follicular fluid, yet the Spanish survey by López-Lería et al. found that 19.6% of embryologists never wore gloves when working with follicular fluid, representing a significant safety gap (33, 34); cryopreservation poses a risk as liquid nitrogen can serve as a vector for cross-contamination if infected specimens leak during storage, with documented cases of HBV transmission through contaminated cryogenic storage (35); and waste handling of contaminated consumables presents sharps injury risk and exposure potential. The prevalence of blood-borne pathogens among fertility patients generally reflects that of the general population, though certain subgroups including men who have sex with men, intravenous drug users, and individuals from high-prevalence geographic regions may have higher rates (36–38). Importantly, many infected individuals are unaware of their status, and the “window period” for seroconversion means that recent infections may not be detected by routine screening, underscoring the need for universal precautions regardless of patient test results (39). Despite clear recommendations for such precautions, adherence to personal protective equipment (PPE) among embryologists remains inconsistent; the Spanish survey revealed that 11.2% of embryologists never wore gloves when handling semen, 19.6% never wore gloves when working with follicular fluid, and 30% rarely or never used protection when working with liquid nitrogen (33, 34). These gaps in PPE adherence may reflect discomfort with glove use during fine manipulation, perceptions of low risk, or inadequate training, but they represent significant vulnerabilities for occupational infection (40).

#### Physical hazards

**Figure 3:**
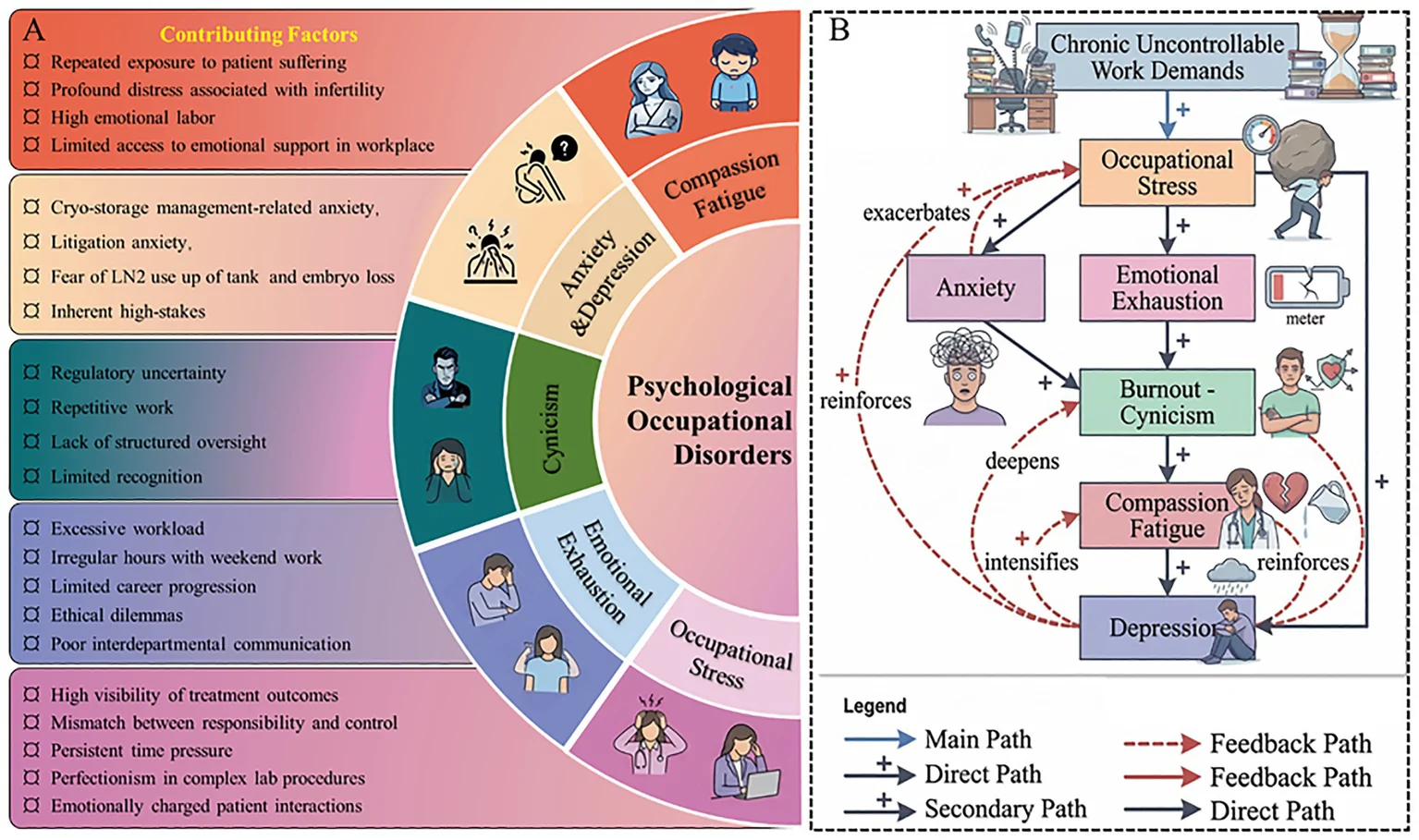
Causal pathway of psychological occupational disorders of clinical embryologists. Multidimensional contributing factors and symptoms: this panel elucidates the hierarchical architecture of occupational hazards and their resultant psychological manifestations. The central semi-circle delineates five core occupational disorders: occupational stress, emotional exhaustion, cynicism, anxiety and depression, and compassion fatigue. Surrounding these outcomes, peripheral modules categorize specific contributing factors.

Additional physical hazards in the embryology laboratory include liquid nitrogen injuries, sharps injuries, and radiation exposure. Cryogenic burns from liquid nitrogen contact represent a specific occupational hazard, as the extreme cold (−196 °C) causes immediate tissue freezing upon contact, with severity depending on exposure duration and surface area; the UK survey identified liquid nitrogen injuries as occurring in more than 3% of respondents (2). Sharps injuries from ICSI or biopsy pipettes, and other sharp instruments pose both infectious disease risk and local trauma (41), and while the small gauge of ICSI or biopsy pipettes may reduce inoculum volume, it does not eliminate infection risk. Radiation exposure is not a significant concern in most embryology laboratories; however, some facilities utilize laser systems for assisted hatching or biopsy, presenting potential eye hazards if improperly shielded Figure 3.

#### Chemical exposure

Embryology laboratories utilize various chemicals with potential occupational health implications, including culture media components which, while generally considered safe, may cause skin or respiratory irritation with repeated exposure; cryoprotectants such as dimethyl sulfoxide (DMSO), ethylene glycol, and propylene glycol used for vitrification, which can penetrate intact skin and may have systemic effects with chronic exposure (34, 42, 43); cleaning and disinfecting agents required for rigorous laboratory protocols that may release volatile organic compounds or cause respiratory sensitization; and carbon dioxide from incubator gas cylinders or centralized systems, which poses asphyxiation risks in confined spaces, though modern laboratories typically have adequate ventilation. The extent to which chronic low-level chemical exposure affects embryologist health remains poorly studied, representing an important knowledge gap.

### Psychological occupational diseases

In addition to physical hazards, embryologists face distinct psychological risks arising from the unique demands of their profession. Unlike many biomedical laboratory roles where the work product remains abstract or removed from clinical outcomes, embryologists operate at the direct intersection of technical precision and patient care, bearing immediate responsibility for the success or failure of fertility treatments. This convergence of high-stakes outcomes, relentless time pressure, and emotional engagement with patient suffering creates a psychological burden that has been systematically documented across multiple studies (4, 5, 44). The international survey by Murphy et al. assessed perceived stress using the validated Perceived Stress Scale (PSS) and found that embryologists in both the UK and US reported moderate stress levels (4).

Multiple factors contribute to embryologist stress, beginning with outcome visibility: unlike many laboratory scientists whose work products are numerical results, embryologists witness the direct consequences of their efforts (or failures) through pregnancy outcomes communicated by clinicians, leading to heightened emotional investment and psychological burden (45). This is compounded by the imbalance between high responsibility and limited control, as embryologists bear substantial responsibility for cycle outcomes yet have limited control over patient factors, ovarian response, or sperm quality that fundamentally influence success, a well-established occupational stressor (46). Time pressure further exacerbates stress, as the biological clock governing ART cycles creates inexorable deadlines: oocytes must be inseminated within hours, fertilization checks cannot be deferred, and embryo transfers occur on scheduled dates regardless of laboratory workload. The demands of perfectionism also play a critical role, as the stakes of embryology work require near-perfect performance, minor errors such as a slightly delayed transfer or momentary temperature fluctuation can compromise outcomes, creating a state of chronic vigilance (4, 10, 47). Finally, patient interactions add emotional labor to technical demands; while embryologists have less direct patient contact than clinicians, they may engage with anxious patients during procedures such as semen collection instruction or transfer counseling, further contributing to psychological strain (48).

Stress among embryologists manifests not only psychologically but also physically, with the Murphy study assessing somatization using the Patient Health Questionnaire (PHQ-15) and finding low to moderate somatic symptom severity among both UK and US embryologists (4). Common somatic complaints included fatigue and low energy, sleep disturbances, headaches, gastrointestinal symptoms, and muscle tension and pain-symptoms that reflect the physiological burden of chronic stress activation and may compound the physical occupational hazards discussed previously.

Burnout has emerged as a critical occupational health concern for embryologists, with prevalence rates exceeding those of many other healthcare professions. Several studies have specifically compared burnout levels among different health care professionals working in ART, and they consistently demonstrate that embryologists report the highest levels of burnout. For instance, Urteaga and Díaz compared burnout levels across ART professional roles using the Maslach Burnout Inventory (MBI-HSS). Their findings revealed that, across all three subscales, embryologists exhibited the highest percentages of burnout when compared to gynecologists, psychologists, nurses, and nurse assistants. In contrast, psychologists showed the lowest levels of emotional exhaustion and depersonalization, while gynecologists reported the lowest levels of reduced personal accomplishment (49). Another landmark study by Murphy et al., using the Maslach Burnout Inventory-General Survey (MBI-GS), assessed three dimensions of burnout among embryologists (4). Emotional exhaustion, feelings of being emotionally overextended and depleted, was extraordinarily high, with 59% of UK embryologists and 62% of US embryologists scoring high on this dimension, indicating a workforce under significant strain (4). Cynicism (or depersonalization), characterized by negative or detached responses to the job, showed notable geographic variation: UK embryologists had lower cynicism scores (43% high) compared to their US colleagues (60% high), a difference attributed to the greater regulatory structure and certainty provided by HFEA (4). Reduced professional efficacy, the third dimension representing diminished feelings of competence and achievement, was also reported, though data were less prominently detailed. Basar et al. confirmed these findings, reporting that embryologists experience the highest emotional burnout levels among all ART professionals, a risk that likely reflects the combination of technical demands, outcome responsibility, and limited professional recognition characterizing embryology practice (5). Several factors have been identified as contributors to embryologist burnout, including excessive workload, with 80–81% of embryologists reporting overtime work (4); irregular hours on weekends and holidays that disrupt work-life balance and social connections (21); limited career progression opportunities, particularly in smaller laboratories or regions without defined career ladders (5); ethical dilemmas involving embryo disposition, multi-fetal pregnancy reduction, or morally challenging cases that create moral distress (50); and poor interdepartmental communication with clinical colleagues, adding interpersonal stress to technical demands (5). The consequences of embryologist burnout extend beyond individual suffering to affect laboratory quality and patient outcomes: burnout impairs attention, memory, and executive function essential for precise, error-free work (51, 52); fine motor skills required for ICSI, biopsy, and other micromanipulation procedures may deteriorate with burnout-related fatigue and distraction (5); burnout increases the likelihood of shortcuts or deviations from protocols, potentially compromising quality and safety; high-burnout laboratories show increased witnessing discrepancies, failures to properly verify patient identity and specimen tracking representing a serious patient safety risk (5); and burnout affects communication quality and patient interactions, reducing patient satisfaction with care (53).

While less systematically studied than burnout, anxiety and depression affect a substantial minority of embryologists; the UK survey found that 27.8% of respondents reported stress and mental health problems, making psychological morbidity the second most common occupational health issue after musculoskeletal disorders (2). A specific anxiety source identified in recent research relates to cryostorage management, with Murphy et al. finding that increasing levels of anxiety about performing cryostorage tasks showed dose-dependent associations with increased risk of burnout on at least two MBI-GS dimensions (in UK embryologists) and PSS and PHQ-15 scores (in both UK and US groups) (4). Anxiety among embryologists is increasingly linked to cryo-storage responsibilities. High-profile incidents of tank failures and specimen losses have heightened concerns about disaster prevention (54). This is compounded by fear of litigation, as malpractice claims involving ART particularly those related to lost or damaged frozen embryos have risen (55); the potential for career-ending legal action adds considerably to the psychological burden of embryology practice.

Embryologists work closely with patients experiencing infertility a condition associated with significant psychological distress, and this repeated exposure to patient suffering can lead to compassion fatigue and secondary traumatic stress, conditions characterized by reduced empathic capacity and intrusive thoughts about patients’ trauma (56). While less studied in embryology than in nursing or mental health professions, the emotional demands of fertility care suggest that compassion fatigue likely affects embryologists as well.

The psychological hazards are as diverse and significant as the physical risks inherent in their work. From the chronic, moderate stress driven by outcome visibility and time pressure, to the higher rates of emotional exhaustion that characterize burnout, the mental health burden on this workforce is profound. This burden is further compounded by specific anxieties related to cryo-storage responsibilities and litigation, as well as the potential for compassion fatigue from engaging with patient suffering. Crucially, these psychological stressors are not merely matters of individual well-being; they have demonstrable consequences for laboratory quality and patient safety, impairing cognitive and psychomotor performance, increasing protocol deviations, and compromising patient interactions.

### Ergonomic hazards and working conditions

**Figure 4:**
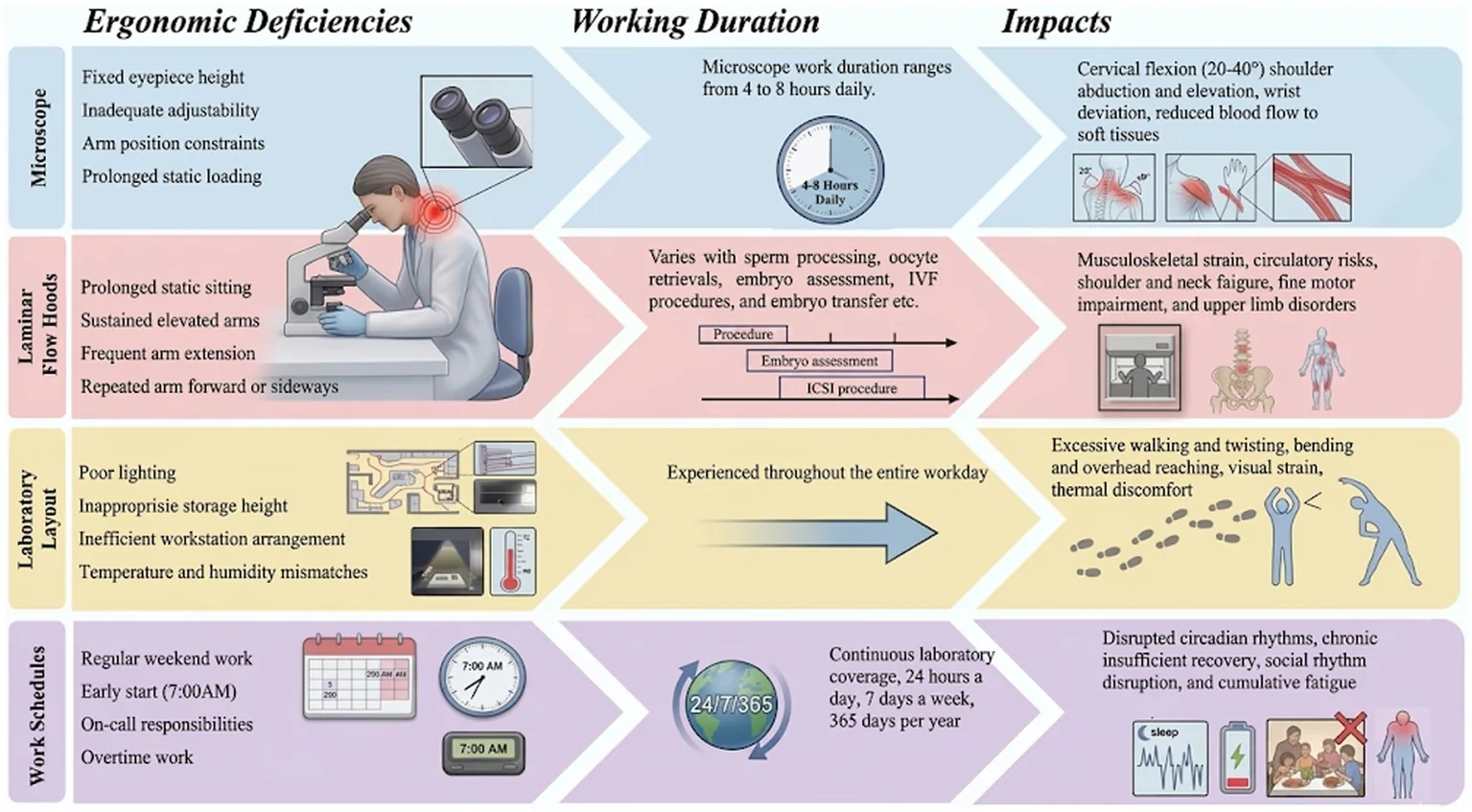
Ergonomic Deficiencies in IVF Laboratory Practice. This matrix systematically outlines ergonomic deficiencies across key IVF laboratory workstations including microscope operation, laminar flow hood use, laboratory layout, and work scheduling, along with exposure durations and their associated physical impacts. It highlights cumulative strain resulting from prolonged static postures, repetitive tasks, and the demands of continuous 24/7 operations.

The physical demands of embryology work are largely shaped by equipment design, laboratory layout, work schedules, and task structures. Figure 4 illustrates the ergonomic deficiencies, exposure durations, and their subsequent impacts across key domains of embryology practice. Traditional microscope configurations, laminar flow hoods, and cryo-storage systems often impose static postures, repetitive movements, and sustained physical strain that contribute to musculoskeletal disorders and fatigue. Similarly, irregular work schedules and the need for continuous laboratory coverage disrupt circadian rhythms and recovery, compounding physical and psychological burden Table 3.

**Table 3:** Consequences of psychological occupational disorders.

Psychological condition	Prevalences	Contributing factors	Consequences
Occupational stress	Embryologists report moderate stress levels (PSS) across the UK and US, with higher anxiety specifically linked to cryo-storage management (4).	Occupational stress arises from outcome visibility, responsibility-control mismatch, time pressure, perfectionism demands, and emotionally charged patient interactions.	Common work-related symptoms include fatigue, sleep disturbances, headaches, gastrointestinal issues, and muscle tension/pain.
Burnout-emotional exhaustion	High emotional exhaustion affects 59% of UK and 62% of US embryologists, the highest rate among ART professionals (4, 5), driven primarily by frequent overtime (80–81%) and cryo-storage-related anxiety.	Emotional exhaustion is driven by excessive workload, irregular hours (including weekends), limited career progression, ethical dilemmas, and poor interdepartmental communication.	Emotional exhaustion consequences: reduced attention/memory, impaired executive function, deteriorated fine motor skills (ICSI/biopsy), lower adherence, more witnessing errors (5), and reduced patient satisfaction.
Burnout-cynicism	Cynicism affects 43% of UK and 60% of US embryologists, a significant geographical disparity attributed to the protective influence of the HFEA regulatory structure in the UK (4).	Cynicism and depersonalization are driven by regulatory uncertainty, lack of structured oversight, repetitive work, and limited recognition.	Cynicism and depersonalization present as detachment, negative attitudes, reduced empathy, and interpersonal difficulties.
Anxiety and depression	Approximately 27.8% of embryologists have anxiety and depression, driven by cryostorage-related task and escalating litigation concerns in ART (2, 4, 39).	Major anxiety and depression contributors include cryostorage-related anxiety (4), litigation anxiety (39), fear of tank failures and specimen loss (55), and the inherent high-stakes consequences of embryology work.	Anxiety and depression correlate with dose-dependent burnout risk, elevated PSS/PHQ-15 scores, and impaired concentration (4).
Compassion fatigue/secondary traumatic stress	Compassion fatigue and secondary traumatic stress remain understudied in embryology, though patterns parallel those in nursing and mental health professions (56)	Compassion fatigue and secondary traumatic stress are driven by repeated patient suffering exposure, infertility-related distress, high emotional labor for professional composure, and limited workplace emotional support.	Compassion fatigue and secondary traumatic stress present as reduced empathy, intrusive thoughts, and emotional numbing.

ART, Assisted reproductive technology; PSS, Perceived stress scale; PHQ, Patient health questionnaire.

#### Microscopes

As the primary tool of embryologists, the microscope poses the most significant ergonomic challenge, as traditional designs force users into postures that violate ergonomic principles. Standard microscopes present multiple ergonomic problems, including fixed eyepiece height that cannot accommodate users of varying statures requiring tall individuals to stoop and shorter individuals to elevate their chairs and adopt shoulder elevation (29, 57); inadequate adjustability, as few modern microscopes provide the range needed for optimal posture despite some offering adjustable eyepiece angles and interpapillary distance; arm position constraints where stage controls require specific positions that may force shoulder abduction and wrist deviation; and prolonged static loading requiring sustained muscle contraction to maintain position, which reduces blood flow and accelerates fatigue. Several interventions can improve microscope ergonomics, beginning with adjustable seating-chairs with full adjustability in seat height, backrest position, and armrests allow embryologists to achieve neutral postures. Monitor-based microscopy systems that display images on adjustable monitors enable more neutral head and neck positions than traditional eyepieces (58). Additionally, while the widely cited 20–20-20 rule is often recommended as a treatment for eye strain, this method does not actually reduce eye strain in practice (59, 60). Instead, only task rotation alternating between microscope work and other laboratory tasks, can effectively reduce cumulative exposure to static postures.

#### Laminar flow hoods

Laminar flow hoods, essential for maintaining sterility during oocyte and embryo handling, often imposes significant physical strain on operators, primarily through prolonged static sitting and sustained unnatural arm postures. Prolonged static sitting during extended procedural sessions (e.g., 2–4 h) contributes to musculoskeletal issues such as lower back pain and cervical spine strain, circulatory problems including venous stasis and elevated risk of deep vein thrombosis (DVT), and adverse metabolic impacts from reduced muscle activity. Concurrently, the technical demands of the work necessitate sustained elevated arm positioning and frequent arm extension. Holding arms above heart level places a continuous load on the shoulder and neck musculature (e.g., trapezius, deltoid), leading to fatigue, tendonitis, or shoulder impingement syndrome, which can compromise fine motor control and precision in delicate procedures like ICSI. Furthermore, repeatedly reaching forward or sideways strains nerves and joints, increasing the risk of upper limb disorders such as thoracic outlet syndrome, median nerve compression, epicondylitis, or carpal tunnel syndrome. This combination of sedentary posture and repetitive upper limb strain creates a cumulative “double burden,” accelerating the onset of work-related musculoskeletal disorders (WRMSDs) among embryologists.

#### Laboratory layout

The physical layout of embryology laboratories affects both workflow efficiency and ergonomic risk: workstation arrangement designed without ergonomic consideration may require excessive walking, twisting, or reaching to access equipment and supplies; storage at inappropriate heights forces bending or overhead reaching for frequently used items; and inadequate or poorly positioned lighting increases visual strain, potentially encouraging awkward postures as embryologists lean to see better. Additionally, while temperature and humidity are strictly controlled for optimal embryo development, these conditions may not align with the thermal comfort of workers, particularly those wearing gowns and gloves.

#### Work schedules and fatigue

Beyond physical layout, the irregular work schedules required for ART practice contribute significantly to occupational fatigue, with implications for both health and performance. Embryologists regularly work weekends for retrievals, ICSI, and transfers, disrupting normal social rhythms and recovery time; oocyte retrievals often begin early typically 7–8 a.m., following patient ovulation trigger approximately 36 h prior, requiring starts before typical business hours; smaller laboratories may require on-call responsibilities for weekend or holiday procedures, further extending workweeks and limiting recovery. Alarmingly, with 80–81% of embryologists reporting overtime work (4), this chronic insufficient recovery time likely contributes substantially to both physical and psychological morbidity.

Mitigating embryologists’ occupational risks requires a multifaceted strategy addressing physical, psychological, and organizational dimensions. Interventions must be tailored to the unique constraints of the ART laboratory specifically its reliance on microscopy, sterile standing requirements, and time-critical workflows. Redesigning equipment, optimizing layouts, and restructuring processes are essential to reduce ergonomic strain and safeguard both staff well-being and patient care quality.

## How to mitigate the occupational health risks for embryologists

Mitigating occupational health risks in embryology requires a multidimensional framework integrating individual behaviors, laboratory practices, organizational policies, and technological innovations. Given the complex interplay of ergonomic, psychological, and physical hazards, no single intervention is sufficient. Instead of relying on piecemeal solutions, meaningful systemic improvement stems from a multi-level framework. It integrates individual focus on ergonomics and PPE with laboratory advancements in safety culture and staffing. At the organizational level, it demands robust regulatory and compensation standards, while technological breakthroughs such as automation and remote monitoring, serve to reduce the cognitive burden on personnel. Table 4 summarizes these strategies into a framework that identifies specific interventions, and their targeted hazards to guide systematic occupational health improvement in ART laboratories.

**Table 4:** Multilevel interventions for enhancing occupational health of embryologists.

Intervention level	Specific strategies	Targeted hazards/conditions	Implementation barriers	Evidence base
Individual	PPE compliance training (61); Ergonomic glove selection (77); Body mechanics & stretching programs (33, 34); Mindfulness-based stress reduction (MBSR) (63); Cognitive-behavioral skills; Peer support networks.	Biological hazards; MSDs; Ocular strain; Occupational distress; Burnout.	Tactile sensitivity issues; Time constraints; Variable individual motivation; Lack of specialized training access.	PPE efficacy in healthcare; Stretching programs recommended (33, 34); Mindfulness efficacy in clinical staff (63).
Laboratory	Ergonomic equipment investment (29, 58); Height-adjustable workstations; Task rotation & equitable scheduling; Evidence-based staffing ratios (64); Non-punitive incident reporting; Regular safety audits.	MSDs; Ocular/auditory strain; Chemical/physical hazards; Chronic workload stress; Burnout.	Capital expenditure; Facility space constraints; Staffing shortages; Organizational inertia in cultural transformation	Vienna consensus on staffing (64); Digital microscopy for ergonomic relief (58); Safety culture paradigms.
Organizational	Regulatory staffing standards (9); Mandated safety programs; Burnout assessment integration (5); Defined career trajectories (9); Fair overtime compensation & wellness benefits (4).	Systemic burnout; Anxiety/Depression; Career dissatisfaction; High turnover intention.	Regulatory latency; Financial constraints; Lack of industry benchmarking; Competing corporate priorities.	HFEA regulatory framework benefits (4); Relation between salary and job satisfaction (4).
Technological	Automated vitrification (67); Robotic cryo-management (4, 67); AI-driven morphology assessment (68–71); Electronic witnessing systems (74, 75); Remote monitoring.	Repetitive task MSDs; Cryo-storage anxiety (4); High cognitive load; Human error risk; After-hours burden.	High initial ROI latency; Complex validation requirements; Steep learning curves; Regulatory hurdles.	Electronic witnessing reduces error rates (74, 75); AI reduces microscope bench time (68–71); Remote monitoring alleviates anxiety (4).

MSD, Musculoskeletal disorder; ROI, Return on investment; HFEA, Human fertilisation and embryology authority; AI, Artificial intelligence.

At the individual level, several interventions can help embryologists mitigate occupational risks. Improving PPE adherence requires addressing both behavioral and practical barriers: regular education and training on transmission routes, infection consequences, and the rationale for universal precautions can improve compliance, as many embryologists underestimate infection risks or overestimate their ability to avoid exposure (61); selecting appropriately sized, thin, powder-free gloves can minimize resistance to glove usage for fine manipulation due to concerns about reduced tactile sensitivity, and double-gloving with an inner indicator glove provides protection while maintaining sensitivity (62); ensuring PPE is readily available at all workstations, as compliance suffers if gloves, gowns, or eye protection require walking to distant supply areas; and laboratory leadership consistently modeling appropriate PPE use, as subordinates are likely to imitate supervisors who disregard precautions. Ergonomics training and body mechanics instruction can also reduce musculoskeletal disorder risk by teaching posture awareness to help embryologists identify neutral versus non-neutral postures and make real-time corrections, providing instruction on proper microscope setup including eyepiece height, interpupillary distance, and stage position during orientation and periodic refresher training, implementing brief stretching breaks between procedures to reduce muscle tension and improve circulation (33, 34), and encouraging general physical fitness, particularly core strength and upper body conditioning, to increase resilience to ergonomic stressors. Additionally, stress management and resilience training can help embryologists cope with occupational stress through mindfulness-based stress reduction programs that have shown efficacy in reducing healthcare worker stress and burnout (63), cognitive-behavioral skills training in cognitive restructuring, problem-focused coping, and adaptive thinking patterns, work-life boundary management encouraging clear boundaries between work and personal life including limiting after-hours email and communication, to support recovery, and fostering collegial peer support networks within laboratories to provide informal psychological first aid.

At the laboratory level, comprehensive interventions encompassing ergonomic equipment, staffing management, and safety culture can substantially reduce occupational health risks. Investing in ergonomically designed equipment encompasses height-adjustable workstations that enable neutral postures for various statures, advanced microscopes featuring tilting heads and remote controls to minimize physical strain, and digital microscopy systems offering maximum flexibility through monitor-based viewing (58). This investment further extends to anti-fatigue matting for standing work to reduce lower extremity strain, optimized storage placing frequently used supplies within easy reach to minimize repetitive bending, and supplemental adjustable task lighting at workstations to reduce eye strain and prevent awkward leaning. Adequate staffing and workload management are equally essential, requiring evidence-based staffing ratios as recommended by the Vienna consensus and other guidelines based on cycle volume although many laboratories currently operate below these recommendations (64); task rotation through different activities such as morning checks, ICSI, and cryopreservation to reduce cumulative exposure to specific ergonomic stressors; schedule optimization by distributing weekend and holiday duties equitably and providing adequate recovery time between on-call periods to support work-life balance; and monitoring and limiting overtime hours to protect against chronic fatigue, even though ART cycles cannot always be scheduled during business hours. Finally, building a robust safety culture through clear, evidence-based standard operating procedures for all activities, including PPE use and emergency response provides guidance and reduces uncertainty; non-punitive incident reporting systems encourage identification of hazards and near-misses before they cause harm; periodic safety audits including direct observation of PPE use and ergonomic assessment identify improvement opportunities; and multidisciplinary safety committees that include embryologist representation ensure that occupational health receives ongoing attention.

At the organizational level, interventions encompassing regulatory standards, professional development, and compensation structures are essential for addressing the systemic factors that contribute to embryologist occupational health risks. Regulatory and accreditation bodies can drive improvements by implementing workforce standards that mandate adequate staffing levels and competency assessment, which indirectly support embryologist well-being (9); establishing safety requirements that require documented safety programs, PPE availability, and hazard communication to create organizational accountability; and integrating burnout assessment into routine quality assurance and regulatory monitoring that would elevate well-being to a quality metric comparable to pregnancy rates or laboratory key performance indicators (KPIs) (5). Addressing the limited career progression that contributes to burnout requires organizational commitment to defined career ladders establishing clear pathways for advancement including senior embryologist, supervisor, and director roles to provide motivation and recognition; supporting embryologist pursuit of professional certification through bodies such as the American Association of Bio-analysts (AAB) or equivalent organizations to validate expertise and create advancement opportunities (9); funding attendance at scientific meetings and professional development courses to demonstrate organizational investment in embryologist growth; and encouraging embryologist participation in research and publication to provide intellectual stimulation and professional recognition. Fair compensation and benefits also play a critical role, as the finding that UK embryologists report better overtime compensation than US colleagues suggests that fair pay for extra hours matters for satisfaction and stress (4); ensuring competitive salaries with other laboratory science positions reduces financial stress and turnover; and providing comprehensive wellness benefits including mental health coverage, gym memberships, and wellness programs supports overall health.

Technological innovations are increasingly offering solutions to reduce embryologist workload and mitigate occupational health risks. Automation and robotics, including automated dish preparation (65, 66) and vitrification systems for embryo cryopreservation (67), can reduce the repetitive stress of manual procedures; automated cryo-storage management systems that track inventory, monitor tank conditions, and retrieve specimens decrease both the physical demands and the anxiety associated with cryo-storage oversight (4); digital imaging and artificial intelligence enable automated embryo assessment, potentially reducing the time embryologists spend at microscopes for morphological evaluation (68–71); and time-lapse imaging integrated into incubators minimizes the need to remove embryos from culture for assessment, thereby decreasing the time of embryo handling and microscope usage (71–73). Emerging laboratory information systems further reduce cognitive load and error risk through electronic witnessing using barcode or radio frequency identification (RFID)-based systems that lessen the cognitive burden of manual verification and alleviate stress about patient identification errors (71, 74, 75); workflow management tools that track procedures, timers, and tasks to reduce the mental load of managing complex schedules (71, 75); and decision support algorithms that flag out-of-range parameters or suggest optimal procedures, reducing uncertainty and supporting consistent practice. Additionally, emerging models of remote and tele-embryology may offer greater flexibility, with remote monitoring systems allowing embryologists to oversee incubators, cryo-storage, and laboratory conditions from off-site locations, thereby reducing the burden of after-hours checks (76), while tele-consultation for complex cases could help distribute expertise and reduce professional isolation in smaller laboratories.

## Research gaps and future directions in embryologist occupational health

Current understanding of embryologist occupational health rests on limited cross-sectional surveys, necessitating research that addresses several critical gaps. Longitudinal studies are essential to establish causal links between workplace exposures and health outcomes, particularly for cumulative musculoskeletal disorders. Furthermore, international comparisons using standardized surveys would elucidate how regulatory frameworks, laboratory practices, and cultural factors influence well-being. Supplementing self-reports with objective metrics such as physical examinations, validated psychological assessments, and biomarker analyses, would substantially strengthen the evidence base for future interventions.

Beyond descriptive studies, the lack of rigorously evaluated interventions highlights a pressing need for experimental research. Ergonomic trials comparing traditional and optimized equipment would provide a basis for evidence-based investment. Similarly, randomized controlled trials targeting stress management, scheduling, or organizational changes are required to identify effective burnout mitigation strategies. Research into behavioral interventions is also vital to improve PPE compliance and inform safer program designs.

As new technologies emerge, their occupational implications require scrutiny. Research should investigate whether automation genuinely reduces hazards or merely transforms them, such as by trading physical strain for increased mental monitoring demands. The impact of AI-assisted decision-making on job satisfaction, professional identity, and cognitive load also warrants investigation. Additionally, tele-embryology pilot studies should assess both operational feasibility and its impact on practitioner health.

Translating these findings into practice requires broad policy engagement. Professional organizations should advocate for regulatory standards that incorporate workforce well-being into formal laboratory oversight. Evidence-based guidelines, analogous to existing laboratory quality protocols should be developed to provide clear, actionable frameworks. Finally, workforce planning based on supply-and-demand projections must inform training capacity to prevent the shortages that exacerbate workload stress.

## Conclusion

As global ART cycles increase and laboratory methodologies grow more complex, embryologists face unprecedented technical and psychological demands—yet their occupational health remains critically overlooked. The evidence is alarming that nearly 50% suffer from musculoskeletal disorders, over 25% have mental health challenges linked to occupational stress, and burnout affects more than 60% in some cohorts. Safety gaps persist, including inconsistent PPE use and cryostorage-related anxiety. Embryologist burnout and stress correlate directly with reduced protocol adherence and increased witnessing errors, ultimately compromising patient safety and clinical outcomes.

To mitigate the aforementioned risks and safeguard embryologist well-being, the following actions are essential. Laboratory directors must implement sustainable staffing models, and mental health support to reduce burnout, musculoskeletal disorders, and safety gaps among embryologists. Meanwhile, regulatory authorities should mandate occupational health standards within ART accreditation frameworks, explicitly linking embryologist well-being to routine quality metrics. Furthermore, with the increasing adoption of automation and artificial intelligence (AI) technologies in IVF laboratories, embryologists must embrace novel technologies and self-monitoring practices, as protecting their well-being is inextricably linked to treatment safety and clinical outcomes.

## Glossary

AAB: American association of bio-analysts
AI: Artificial intelligence
ART: Assisted reproductive technology
ASRM: American society for reproductive medicine
CAP: College of American pathologists
DMSO: Dimethyl sulfoxide
ESHRE: European society of human reproduction and embryology
EUTCD: EU tissues and cells directive
HBV: Hepatitis B virus
HCPC: Health and care professions council
HCV: Hepatitis C virus
HEPA: High-efficiency particulate air
HFEA: Human fertilisation and embryology authority
HIV: Human immunodeficiency virus
ICSI: Intracytoplasmic sperm injection
IVF: *In vitro* fertilization
KPI: Key performance indicator
MBI-GS: Maslach burnout inventory-general survey
MBSR: Mindfulness-based stress reduction
MSD: Musculoskeletal disorder
PHQ: Patient health questionnaire
PPE: Personal protective equipment
PSS: Perceived stress scale
RFID: Radio frequency identification
ROI: Return on investment
TJC: The joint commission
WRMSD: Work-related musculoskeletal disorder
