# Correction: Prioritizing embryologist health: occupational health hazards and preventive strategies

**DOI:** 10.3389/fpubh.2026.1926975

**Published:** 2026-08-10

**Authors:** Man-Xi Jiang, Shao-Qing Chen, Huai L. Feng, Chang-Long Xu, Yan Zhu

**Affiliations:** 1 Center for Reproductive Medicine, The Affiliated Guangdong Second Provincial General Hospital of Jinan University, Guangzhou, China; 2 New York Fertility Center, New York-Prebyterian Healthcare System Affiliate Weill Cornell Medical College, New York, NY, United States; 3 Guangxi Clinical Research Center for Reproductive Medicine, Nanning Second People's Hospital/The Third Affiliated Hospital of Guangxi Medical University, Nanning, China; 4 Department of Immunology, University of Pittsburgh, Pittsburgh, PA, United States

**Keywords:** burnout, clinical embryologist, ergonomics, laboratory safety, musculoskeletal disorders, occupational health, preventive strategy

The reference for [58] was erroneously written as “Frone MR, Tidwell M-CO. The meaning and measurement of work fatigue: development and evaluation of the three-dimensional work fatigue inventory (3D-WFI). J Occup Health Psychol. (2015) 20:273–88. doi: 10.1037/a0038700”. The correct reference is “Yu D, Green C, Kasten SJ, Sackllah ME, Armstrong TJ. Effect of alternative video displays on postures, perceived effort, and performance during microsurgery skill tasks. *Appl Ergon*. (2016) 53:281–9. doi: 10.1016/j.apergo.2015.10.016”.

The reference for [64] was erroneously written as “of Embryology ESIG. The Vienna consensus: report of an expert meeting on the development of ART laboratory performance indicators. Reprod Biomed Online. (2017) 35:494–510. doi: 10.1016/j.rbmo.2017.06.015”. It should be “ESHRE Special Interest Group of Embryology and Alpha Scientists in Reproductive Medicine. The Vienna consensus: report of an expert meeting on the development of ART laboratory performance indicators. *Reprod Biomed Online*. (2017) 35:494–510. doi: 10.1016/j.rbmo.2017.06.015”.

The original version of this article has been updated.

